# Network-based framework for studying etiology and phenotypic diversity in primary ciliopathies

**DOI:** 10.1186/s13059-025-03899-7

**Published:** 2026-02-19

**Authors:** Ellen M. Aarts, Diederik S. Laman Trip, Ruxandra Neatu, Charlotte G. Martin, Beth Riley, Alison Kraus, Abigail Green, Mohamed H. Al-Hamed, Rachel E. Armstrong, John A. Sayer, Ruxandra Bachmann-Gagescu, Pedro Beltrao

**Affiliations:** 1 Institute of Molecular Systems Biology, ETH Zurich, Zurich, Switzerland; 2 Swiss Institute of Bioinformatics, Lausanne, Switzerland; 3 Bioscience Institute, Newcastle University, Newcastle Upon Tyne, UK; 4 Yorkshire Regional Genetics Service, Chapel Allerton Hospital, Leeds, UK; 5 Department of Clinical Genomics, Center for Genomic Medicine, King Faisal Specialist Hospital and Research Center, Riyadh, 11211 Saudi Arabia; 6 Renal Services, The Newcastle Upon Tyne Hospitals NHS Foundation Trust, Newcastle, UK; 7 NIHR Newcastle Biomedical Research Centre, Newcastle Upon Tyne, UK; 8 Institute of Medical Genetics, University of Zurich, Schlieren, Switzerland; 9 Department of Molecular Life Sciences, University of Zurich, Zurich, Switzerland

## Abstract

**Background:**

Recent advances in sequencing technologies have increasingly enabled the identification of genetic causes for human monogenic diseases. However, systematic understanding remains limited due to the rarity, genetic heterogeneity, and complex genotype–phenotype relationships of these diseases. Primary ciliopathies are a diverse group of rare disorders caused by variants in genes associated with the cilium, a cellular organelle involved in signaling during development and cell homeostasis. These genetic variants result in a wide spectrum of clinical phenotypes involving the brain, eye, kidney, and skeleton. It remains unclear to what extent this phenotypic diversity can be attributed to the disease-causing genes and their specific roles in ciliary function.

**Results:**

Here, we systematically compared human primary ciliopathies with each other and with mouse phenotypes by propagating known disease genes through a network of protein interactions. Network propagation improved the clustering of primary ciliopathies with shared clinical phenotypes and facilitated the identification of mouse phenotypes closely related to primary ciliopathies, due to shared groups of proteins in the interaction network. By leveraging this phenotype-specific approach, we prioritized candidate genes for specific ciliopathies and identified likely pathogenic variants in *CEP43*, a previously unrecognized ciliopathy gene, in three previously unsolved cases.

**Conclusions:**

This study demonstrates that network propagation enhances the genetic and phenotypic understanding of primary ciliopathies, aiding in the prioritization of candidate genes and providing a framework for unraveling shared underlying mechanisms for other rare genetic diseases.

**Supplementary Information:**

The online version contains supplementary material available at 10.1186/s13059-025-03899-7.

## Background

In recent years, decreasing sequencing costs have significantly advanced our understanding of human genetic disorders. However, the precise etiology and molecular mechanisms of many inherited diseases remain elusive. For monogenic diseases, the genetic cause is often identifiable, yet a systematic understanding of disease etiology and its associated phenotypes is frequently lacking. The difficulty lies in the rarity and genetic heterogeneity of these diseases together with difficulties linking the genotype to complex phenotypes. To uncover disease etiology and molecular mechanisms, many studies have employed network-based approaches [1–7]. While these approaches have predominantly targeted common disorders, recent applications have extended to rare disorders too [8–11].

Network-based approaches have been utilized in numerous ways to study pleiotropy [6], drug targets [3], disease genes [2, 12], and molecular mechanisms [8]. A widely adopted method within these approaches is network propagation, which is based on the guilt-by-association principle [13]. In network propagation, disease genes are mapped onto a network of molecular interactions, and information is propagated over the network to enhance our understanding of diseases. With this approach, it is assumed that groups of interacting proteins will tend to have similar cellular roles and result in similar disease phenotypes when mutated. The link between interacting proteins and disease can also help highlight the underlying physiological and pathophysiological molecular mechanisms.

One group of rare monogenic disorders exemplifying the need for systematic studies are primary ciliopathies. Ciliopathies are caused by variants in genes associated with a single organelle, the cilium. Known as the antennas of the cell, cilia are sensory organelles perceiving, transducing, and regulating a large variety of signals, thereby controlling cellular behavior during development and cell homeostasis. As cilia are present on most vertebrate cells, it is not surprising that variants in ciliary genes can result in multi-systemic disorders with symptoms including polydactyly, skeletal dysplasia, brain malformations, retinal disease, or fibrocystic renal disease [14]. What is less intuitive, however, is that despite the widespread expression of ciliopathy genes, many ciliopathies affect predominantly a single organ system. For instance, according to recent classifications, 6 out of 34 primary ciliopathies are associated with a single organ and for secondary ciliopathies (defined by clinical ciliopathy phenotypes but non-ciliary localization of the involved proteins) 5 out of 18 disorders affect a single organ [15]. Moreover, the other primary and secondary ciliopathies are each defined by a unique combination of symptoms, with substantial phenotypic and genetic overlap among them. As a result, efforts to study genotype-phenotype associations have seen limited success so far [16–21]. It remains unclear to what extent the causal genes and their ciliary function(s) can explain the observed phenotypic divergence.

In this study, we systematically investigate rare disease etiology and phenotype-related molecular mechanisms through network propagation. In a previous study, we have shown that network propagation can be used to study the etiology of genome-wide association studies (GWAS)-linked traits [6]. Here, we extend that approach to show that network propagation can also reveal relationships between rare disorders that share similar clinical phenotypes, as well as identify interconnected proteins underlying organ-specific phenotypes. In addition, to expand the limited knowledge on rare diseases, we demonstrate how genetic information from non-human models, specifically mice, can enhance our understanding of ciliopathies. Mouse models have been used extensively to further understand genetic diseases and their molecular mechanisms, making them a valuable source of complementary data [22, 23]. We developed an approach that uses network propagation scores to identify mouse phenotypes relevant to human diseases, allowing us to incorporate these phenotypes alongside propagation scores from the human diseases to enhance candidate gene prioritization. As a proof-of-concept, we apply this approach for ciliopathy-specific disease gene prioritization, resulting in the identification of pathogenic variants in *CEP43* in three patients with ciliopathy phenotypes and previously unsolved genetic diagnoses. Overall, our approach enables systematic comparison of ciliopathies with similar organ involvement and allows for candidate gene prioritization specific to each ciliopathy through the integration of mouse phenotype data and human genetic information.

## Results

### Network-based similarity between ciliopathies with shared clinical phenotypes

**Fig. 1:**
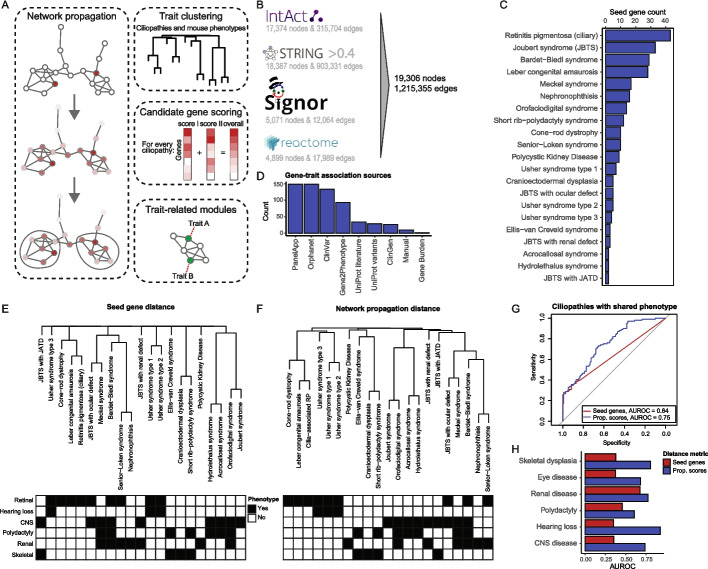
Network propagation improves phenotypic comparisons of ciliopathies. **A** Schematic overview of the approach: network propagation is used to compare traits (ciliopathies and mouse phenotypes), detect protein modules underlying organ-specific phenotypes, and prioritize candidate genes. **B** Number of nodes and edges from Intact, STRING, SIGNOR, and Reactome included in our undirected PPI network. **C** Number of disease genes for each of the 21 selected primary ciliopathies. **D** Number of gene-disease associations from each source of evidence. **E**–**F** Hierarchical clustering of ciliopathies based on overlap of known disease genes (Jaccard distance) (**E**) or on network propagation scores (Euclidean distance) (**F**). Dendrograms are manually annotated with the main clinical organ phenotypes, including retinal disease, hearing loss, CNS disease, polydactyly, renal disease, and skeletal dysplasia. **G** ROC curves comparing recovery of ciliopathy pairs with at least one shared clinical phenotype based on known disease gene overlap (red) or on network propagation scores (blue). Grey line shows a random classifier. **H** AUROC values for the predictions in (**G**), split by the clinical phenotypes. JATD, Jeune asphyxiating thoracic dystrophy

To systematically study ciliopathies, we applied network propagation to cluster traits including human ciliopathies and mouse phenotypes, identify trait-related protein modules, and predict candidate genes (Fig. 1a). For network propagation, we used a previously developed molecular interaction network [6] that integrates both direct (i.e., physical) and indirect protein–protein interactions (PPIs) from Reactome [24], IntAct [25], SIGNOR [26], and STRING [27] (Fig. 1b). To select seed genes for performing network propagation, we collected known disease genes for each human ciliopathy from the Open Targets Platform (OTAR) [28], which aggregates data from several sources such as ClinVar [29], Orphanet [30], and Genomics England PanelApp [31], and we curated the gene-disease pairs. In total, we collected 174 unique disease genes for 21 primary ciliopathies, with the number of genes ranging from 2 to 42 per ciliopathy (Fig. 1c, Additional file 1: Table S1). Most evidence was obtained from Genomics England PanelApp, Orphanet, ClinVar, and Gene2Phenotype [32] (Fig. 1d, Additional file 1: Table S2). Furthermore, we annotated ciliopathies with their main clinical phenotypes according to affected organ systems (Additional file 1: Table S1). Among these, 53 genes were linked exclusively to ciliopathies where a single organ is affected. Since organ phenotypes are largely consistent across ciliopathy disorders and given the significant overlap in clinical phenotypes and disease genes among ciliopathies, we assumed that the underlying pathomechanisms for each organ are similar across disorders. Moreover, published clinical descriptions often lack the detail needed to justify further phenotypic granularity beyond organ involvement. Some ciliopathies have been classified into subtypes based on clinical phenotypes, such as “Joubert syndrome” (JBTS – defined by the presence of a specific brain malformation), “JBTS with ocular defect” (brain malformation and additional retinal involvement), “JBTS with Jeune asphyxiating thoracic dystrophy (JATD)” (brain malformation and additional skeletal dysplasia), and “JBTS with renal defect” (brain malformation and additional renal involvement), which we kept separate. In this study, we associated each disease gene only with the most specific subtype from OTAR, removing associations from the broader type (e.g., associating a gene with “JBTS with ocular defect” rather than with “JBTS”). Additionally, we excluded non-ciliary genes for retinitis pigmentosa (RP), as this disorder can also be caused by many other cellular mechanisms.

To explore whether network propagation can be used to group ciliopathies based on shared underlying mechanisms, we first asked whether disorders with similar clinical phenotypes also have similar network propagation scores. For this, we performed network propagation for each ciliopathy, generating a score for every protein in the PPI network. These scores reflect the proximity of each protein to the disease-associated genes, and similar propagation scores between distinct ciliopathies could suggest the involvement of similar proteins in the diseases. To assess whether network propagation improves clustering of ciliopathies with similar clinical phenotypes beyond gene overlap, we first calculated the overlap in known disease genes between the ciliopathies using the Jaccard distance and generated a hierarchical tree (Fig. 1e, see methods). We then calculated the Euclidean distances between network propagation scores for all ciliopathies and constructed a second tree based on these distances (Fig. 1f). Clustering of ciliopathies using network propagation scores resulted in a hierarchical tree with more depth and branching, allowing for more comparisons between ciliopathies, as ciliopathies without shared disease genes could now be compared to other ciliopathies. To quantify this, we compared Jaccard and Euclidean distances between ciliopathy pairs that share one clinical phenotype and those that do not. We found that the distances based on network propagation scores resulted in better recovery of ciliopathy pairs with a shared clinical phenotype, evaluated by the area under the ROC curve (AUROC) (AUROC = 0.75, 95% CI: 0.69–0.82), compared to the recovery using the overlap of known disease genes (AUROC = 0.64, 95% CI: 0.58–0.70, Fig. 1g). This suggests that known disease genes associated with phenotypically similar ciliopathies are interconnected within our PPI network, reflecting shared underlying molecular mechanisms.

Similarly, we found that the network propagation scores improved the recovery of ciliopathy pairs with one specific shared clinical phenotype (Fig. 1h). Network propagation mainly enhanced recovery of ciliopathy pairs with hearing loss, skeletal dysplasia, and central nervous system (CNS) disease, and minimally enhanced recovery of ciliopathy pairs with renal involvement. The latter ciliopathies already had a higher degree of shared disease genes, minimizing the value of the network propagation. These findings suggest that interconnected proteins may underlie specific organ involvement in ciliopathies, demonstrating some degree of phenotype specificity.

### Protein modules associated with primary ciliopathy phenotypes

To identify groups of highly connected proteins underlying the observed phenotype-specific clustering of ciliopathies, we clustered the PPI network into protein modules using Walktrap clustering [33] (Additional file 1: Table S3). We then linked individual ciliopathies to each protein module if the module contained at least one disease gene from the corresponding ciliopathy and had significantly higher network propagation scores compared to network propagation scores from the full PPI network for that ciliopathy (adjusted *p*-value < 0.05, Wilcoxon Rank Sum test). Protein modules were annotated based on GO enrichment terms.

**Fig. 2:**
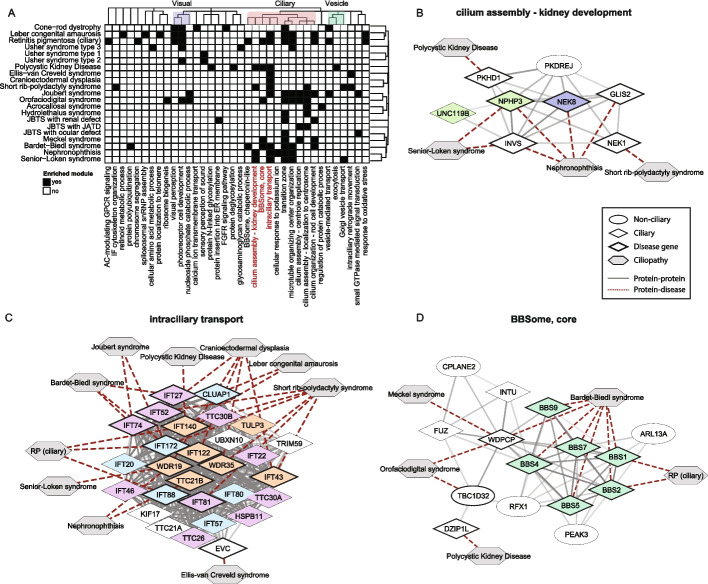
Identification of phenotype-specific protein modules in ciliopathies using network propagation scores. **A** Heatmap showing protein modules with significant enrichment of high network propagation scores for ciliopathies in black (adjusted *p*-value < 0.05, Wilcoxon Rank Sum test). Ciliopathies are clustered by network propagation scores, and protein modules are clustered by re-clustering rounds of protein network clustering. Protein modules describing similar functional terms are highlighted on top: “visual perception” (purple), “ciliary” (red), “vesicle” (green). Protein modules listed in red are detailed in the following panels. **B** Detailed view of the module “cilium assembly – kidney development”. Genes in CiliaCarta and SYSCILIA Gold Standard v2 (SCGSv2) are represented by diamonds, other genes by ellipses, and ciliopathies by grey hexagons. Known ciliopathy genes are outlined in black (as based on initial determination of ciliopathy seed genes). Solid edges represent interactions from Intact or STRING, with transparency indicating the evidence score. Dotted edges indicate gene-disease associations. Nodes are colored based on selected complexes from Boldt et al.: ARL/NPHP/UNC119 (light green) and ANKS/NEK (blue). **C** Detailed view of the module “intraciliary transport”. Node colors by Boldt et al.: IFT-B1 (light blue), IFT-B2 (purple), and IFT-A (orange). **D** Detailed view of the module “BBSome, core”. Node colors by Boldt et al.: BBSome (green). AC, adenylate cyclase; GPCR, G protein-coupled receptor; IF, intermediate filament; FGFR, fibroblast growth factor receptor

We next focused on three protein module clusters assigned with ciliopathy-relevant GO terms, related to cilia, visual process, and vesicle transport (Fig. 2a). We found that the visual protein modules were indeed mainly linked to eye-related ciliopathies, while protein modules in the “ciliary” cluster were linked to a broad range of ciliopathies. However, within the “ciliary” cluster, some specificity was observed with some modules being associated with specific ciliopathies. For example, the protein module “cellular response to potassium ion” contains several NIMMA related kinases (NEK) and Ankyrin Repeat And Sterile Alpha Motif Domain Containing (ANKS) proteins and is exclusively enriched for nephronophthisis. Similarly, the protein module “cilium assembly – kidney development” was primarily linked to the renal ciliopathies Polycystic Kidney Disease (PKD), Senior-Loken syndrome (SLS), and nephronophthisis (NPHP) (Fig. 2b). A separate module also involved in cilium assembly, labeled “cilium assembly – centriole replication” consisted primarily of centrosomal proteins and associated with “pure JBTS” and JBTS with skeletal phenotypes (Jeune) (Additional file 2: Fig. S1a). The fact that KIAA0753 and CEP120, which in our initial seed gene-disease association based on OTAR were associated only with “pure JBTS” (Additional file 1: Table S1), are now close to skeletal phenotypes is consistent with their identification in skeletal ciliopathies in the literature [34, 35], illustrating how this approach can identify true genotype-to-phenotype links. In contrast to this module containing centrosomal proteins associated with a limited number of ciliopathies, the module “microtubule organization center” is very large and is associated with most ciliopathies. The contrast between these modules suggests the existence of stratification among centrosomal proteins with distinct functions.

In addition to modules linked to specific phenotypes, we observed that some ciliopathies, such as cranioectodermal dysplasia (CED) and Usher type 1, were associated with only a single protein module in which their disease genes were highly interconnected. In contrast, disease genes from for example Usher type 3 were distributed across multiple modules (Fig. 2a). This could suggest that Usher type 1 and CED are caused by more specific molecular mechanisms than Usher type 3.

Looking further into the identified protein modules, we found that several modules represented known protein complexes as defined by Boldt et al. [36], such as the BBSome, intraflagellar transport (IFT) complexes, and the transition zone. This serves as a positive control that our approach does identify known protein complexes. As expected, the BBSome was primarily associated with Bardet-Biedl Syndrome (BBS), whereas the intraciliary transport and transition zone modules were associated with many ciliopathies. For skeletal ciliopathies, such as CED and short rib-polydactyly syndrome (SRP), most known disease genes were found in the intraciliary transport module (6/11 for SRP and 5/5 for CED), supporting the idea that defects in this mechanism are main drivers of skeletal phenotypes (Fig. 2c). Interestingly, IFT54 (TRAF3IP1), a core IFT-B component, was not part of this module but instead clustered in the “Golgi vesicle transport” module (Additional file 2: Fig. S1b), aligning with previous findings that it also plays roles outside of intraflagellar trafficking [37]. Furthermore, the transition zone module appeared more broadly associated with ciliopathies than previously suggested. While earlier studies proposed that transition zone dysfunction is primarily linked to the JBTS/MKS/NPHP spectrum [38], our findings also show associations with unrelated ciliopathies, including BBS and retinal disorders (Additional file 2: Fig. S1c).

Lastly, the BBSome module did not only include BBSome complex proteins, but also proteins from the CPLANE complex, consisting of CPLANE2, INTU, FUZ, and WDPCP, and other proteins such as TBC1D32 and DZIP1L (Fig. 2d). Despite enrichment for BBS, we found no link to specific clinical phenotypes via single-organ disorders. In contrast, the module “BBSome, chaperonin-like” was only associated with two ciliopathies with eye-related phenotypes (RP and BBS). Notably, BBS was the only ciliopathy linked to the module “protein polyubiquitination”, driven solely by the disease gene *TRIM32* (Additional file 2: Fig. S1d). *TRIM32* has only been linked to BBS in a single publication [39], while it is also implicated in other disorders [40]. However, several experimental studies have demonstrated a functional link between the BBSome and ubiquitination, supporting the association between BBS and the protein module [41–43].

Together, these observations underscore the ability of our approach to uncover phenotype-specific protein modules, generating hypotheses for further studies into genotype-phenotype associations and general ciliopathy molecular mechanisms.

### Comparison of network propagation between human ciliopathies and comprehensive mouse phenotypes

Having established that network propagation clusters together ciliopathies with similar phenotypes, we sought to apply the same methodology to identify mouse models that resemble human ciliopathies. Mouse models are chosen as they are extensively available for many genes and phenotypes. For example, the Mouse Genome Database includes 17,443 genes with targeted alleles and 74,265 genotypes with phenotype annotations [44]. By linking mouse models to human ciliopathies through related genetics, we wanted to gain mechanistic insights into the organ involvement of ciliopathies and ultimately use the mouse phenotypes for prioritizing novel disease genes for each ciliopathy separately.

We obtained all available 3,524 mouse phenotypes with the human orthologs of their known associated genes from the Mouse Genome Database and propagated the associated genes for each phenotype through the human PPI network independently (Additional file 1: Table S4). For each human ciliopathy, we then selected the twenty mouse phenotypes with the most similar network propagation scores based on Euclidean distances as compared to the network propagation scores obtained for human ciliopathies (see methods) (Additional file 1: Table S5).

The selected mouse phenotypes were found to have little overlap in seed genes with their corresponding human ciliopathy based on the Jaccard distance (Additional file 2: Fig. S2a), although only 8 out of 420 comparisons (20 phenotypes × 21 ciliopathies) had no seed gene overlap at all (Jaccard distance of 1) and at least 8 out of 20 mouse phenotypes per ciliopathy would also have been selected based solely on seed gene overlap (Additional file 2: Fig. S2b). Selection based on network propagation scores was more robust than selection based on seed gene overlap, as the selected mouse phenotypes remained significantly more similar after removing 60% (*p*-value = 0.036; two-sample t-test) and 80% (*p*-value = 0.0091; two-sample t-test) of the seed genes when network propagation was used for selection (Additional file 2: Fig. S2c).

**Fig. 3:**
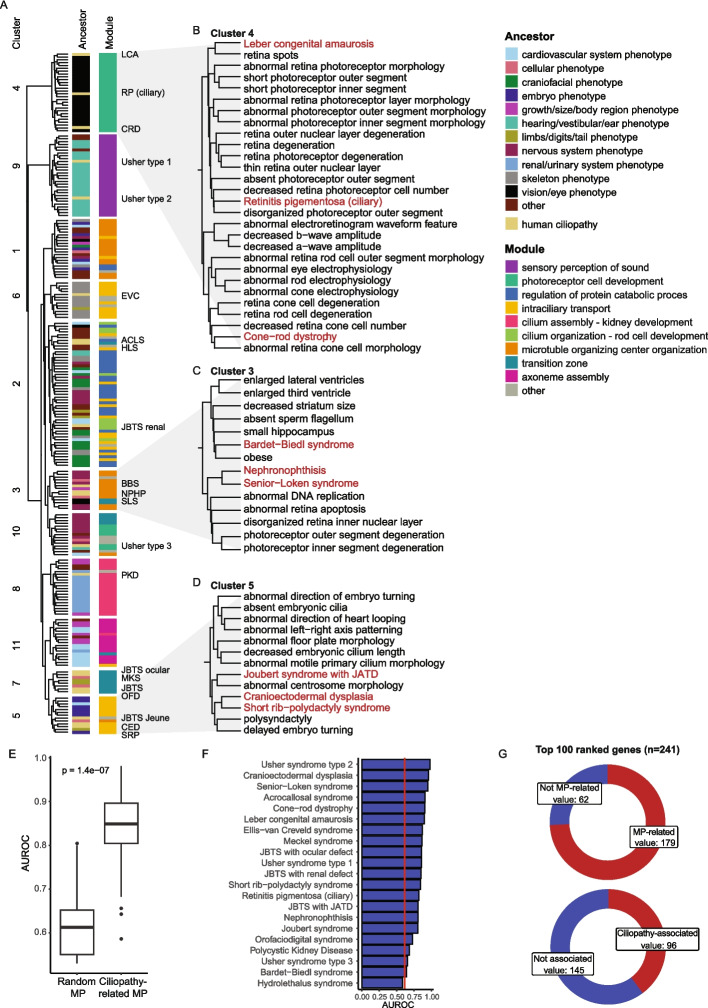
Identification of mouse phenotypes resembling individual ciliopathies through network propagation. **A** Hierarchical clustering of ciliopathies with related mouse phenotypes based on network propagation scores. Mouse phenotypes are annotated by their ancestor terms and all traits are annotated by their associated protein modules. For visualization, traits are colored based on the most represented ancestor term and protein module within each respective cluster. Rows containing human ciliopathies are labeled with their corresponding abbreviations and colored yellow in the ancestor annotation. **B**-**D** Zoom-in views of cluster 4 (**B**), cluster 3 (**C**), and cluster 5 (**D**). Human ciliopathies are in red and mouse phenotypes in black. **E** AUROC scores of known human ciliopathy gene predictions using network propagation scores from ciliopathy-related mouse phenotypes versus randomly selected mouse phenotypes. Ciliopathy disease genes were excluded from the seed genes of mouse phenotypes prior to running network propagation. Genes were ranked for each ciliopathy based on the combined network propagation scores of selected mouse phenotypes versus random mouse phenotypes. Two sample t-test for statistics. **F** AUROC scores from (**E**) visualized per ciliopathy. The red line marks the median AUROC scores when using random mouse phenotypes. **G** Donut plots of the top 100 ranked genes for each ciliopathy by mouse phenotypes indicating whether genes were already associated to those mouse phenotypes (top) or to human ciliopathies (bottom). MP, mouse phenotype; LCA, Leber congenital amaurosis; RP, Retinitis Pigmentosa, CRD, cone-rod dystrophy; EVC, Ellis-van Creveld syndrome; ACLS, acrocallosal syndrome; HLS; Hydrolethalus syndrome; JBTS, Joubert syndrome; BBS Bardet-Biedl syndrome; NPHP, Nephronophthisis; SLS, Senior-Løken syndrome; PKD, Polycystic Kidney Disease; MKS, Meckel syndrome; OFD, orofaciodigital syndrome; CED, Cranio-Ectodermal Dysplasia; SRP, Short-Rib-Polydactyly syndrome

Finally, we clustered the selected mouse phenotypes and human ciliopathies using the network propagation scores and annotated the mouse phenotypes with their broader phenotype categories, termed ancestors, from the Mammalian Phenotype Ontology database [45] (Fig. 3a, Additional file 1: Table S6). We found that mouse phenotypes with similar ancestors generally clustered together. For example, cluster 4 had a high fraction of vision/eye phenotypes, while cluster 6 primarily contained skeletal phenotypes (Additional file 2: Fig. S3a). Importantly, we found that the mouse phenotypes often matched the clinical phenotypes of the human ciliopathies they clustered with. The vision/eye mouse phenotypes in cluster 4, for example, clustered together with eye-related human ciliopathies (Fig. 3b). In contrast, most multi-systemic ciliopathies clustered with mouse phenotypes spanning a broader range of phenotype categories, reflecting their pleiotropic nature. Despite this variability in phenotypes, we found that human and mouse traits often converged on the same underlying protein modules. To identify these links, we calculated the enrichment of network propagation scores from mouse phenotypes in the modules defined by Walktrap clustering on the human PPI network (Additional file 2: Fig. S3b, Additional file 1: Table S7). For instance, cluster 3 had a high fraction of both human and mouse traits with high network propagation scores in the module “microtubule organizing center organization”, containing proteins important for ciliogenesis, but included diverse mouse phenotypes, ranging from obesity to photoreceptor degeneration (Fig. 3c). While diverse, these mouse phenotypes partly matched the clinical phenotypes of BBS (obesity, CNS disease, retinal disease), NPHP (retinal disease), and SLS (retinal disease). Interestingly, one mouse phenotype in this cluster, “small hippocampus”, was genetically close to BBS based on various mouse models but has not yet been established as a clear clinical feature in individuals with BBS, although it was reported in one previous study [46]. Similarly, cluster 5, enriched for the module “intraciliary transport”, grouped skeletal ciliopathies together with a variety of mouse phenotypes, such as polysyndactyly and abnormal centrosome morphology (Fig. 3d). Notably, the latter mouse phenotype shares minimal direct gene overlap with skeletal ciliopathies (only CDK5RAP2) but was still closely related based on the network propagation scores. This suggests that centrosome morphology may be a relevant but underexplored mechanism in skeletal ciliopathies. Overall, this demonstrates that integrating mouse phenotypes via network propagation not only reinforces known clinical associations but also highlights new candidate phenotypes and mechanisms that warrant further functional validation.

We further aimed to investigate if these mouse phenotypes could also be used to propose novel human ciliopathy genes. To test this, we excluded known human ciliopathy genes from the mouse phenotype-associated genes and tried to retrieve the human genes using network propagation with the remaining mouse genes as seed genes (see methods). This worked significantly better when using ciliopathy-related mouse phenotypes compared to random mouse phenotypes (Fig. 3e, *p*-value = 1.4e-7; two sample t-test) and best when using the ten mouse phenotypes with the most similar propagation scores compared to inclusion of more phenotypes (Additional file 2: Fig. S4a). Selecting ciliopathies based on a distance threshold did not improve predictions, as a cutoff of ≥ 180 was required to include mouse phenotypes for all ciliopathies, resulting in AUROC values similar to selecting the top 10 phenotypes (Additional file 2: Fig. S4b). This threshold can still be considered stringent (Additional file 2: Fig. S4c), indicating that the comparable AUROC was not due to inclusion of overly broad phenotype sets. Because the number of seed genes correlated with the number of nearby phenotypes (Additional file 2: Fig. S4d), we proceeded with the top 10 phenotypes to minimize bias. Selecting the top 10 phenotypes based on network propagation, rather than seed gene overlap alone resulted in only a modest improvement in predictions (Additional file 2: Fig. S5a). However, omitting network propagation when integrating the phenotypes and instead simply counting how often a gene appeared as a seed gene for the top 10 phenotypes markedly reduced performance (Additional file 2: Fig. S5b), underscoring the importance of network propagation in this approach.

The effectiveness of gene recovery varied across human ciliopathies, with recovery of Usher type 2 genes performing the best and hydrolethalus syndrome (HLS) genes the worst (Fig. 3f). We found that the combination of mouse phenotypes for predicting human ciliopathy genes identified additional high-scoring genes that were not directly linked to the mouse phenotypes or human ciliopathies (Fig. 3g). For every ciliopathy, we took the top 100 ranked genes, which resulted in a total of 241 unique genes. Of these, 62/241 were not yet associated with the mouse phenotypes used in the analysis and 145/241 were not yet linked to a human ciliopathy. This suggests that integrating multiple mouse phenotypes allows for the discovery of additional potentially relevant disease genes compared to just taking the known genes associated with the separate mouse phenotypes.

### Prioritization of human ciliopathy genes using network propagation scores and disease-related mouse phenotypes

To further improve identification of novel candidate genes for ciliopathies, we combined mouse phenotype-based ranking of genes with disease-specific network propagation scores and gene expression patterns in human tissues. Here, we accounted for biases towards well-studied genes by permuting the network propagation scores 1,000 times using random seed genes while maintaining the same PPI network (Additional file 2: Fig. S6). For the gene expression patterns, we obtained single-cell RNA sequencing data from the Human Protein Atlas (HPA) [47]. Since ciliary genes are typically expressed at low levels, we used relative gene expression to identify cell types with the highest and lowest expression of known ciliopathy genes (Additional file 2: Fig. S7a). Cell types, as defined by HPA, with high expression included photoreceptor cells, astrocytes, spermatocytes, and ciliated cells. By comparing the expression patterns of all genes to those of known ciliopathy genes (see methods), a logistic regression model successfully retrieved ciliopathy genes that were removed for testing (Additional file 2: Fig. S7b) (AUROC = 0.79).

To integrate the three scores for each ciliopathy (permuted network propagation, mouse phenotype ranking, and expression pattern), a logistic regression model was trained on four ciliopathies with the highest numbers of known genes (RP, JBTS, BBS, and Leber congenital amaurosis (LCA)). Because of the limited number of known genes per ciliopathy, we trained the model parameters for the four ciliopathies together. However, the predictions are still ciliopathy specific, as the feature scores are calculated separately for each ciliopathy.

**Fig. 4:**
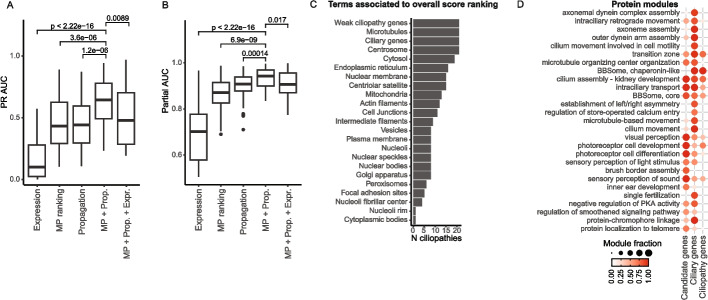
Model for candidate gene prediction specific to each ciliopathy. **A** PR AUC for logistic regression models combining mouse phenotype ranking (MP), permuted network propagation scores (Propagation), and gene expression scores (Expression) in various combinations. **B** Partial AUC (1.00–0.95 specificity) for the same models as in (**A**). **C** Number of ciliopathies (N ciliopathies) with significant GSEA for defined gene sets. Weak ciliopathy genes = genes from databases with low confidence scores. Ciliary genes = genes in CiliaCarta and SCGSv2. Other gene sets are obtained from Human Protein Atlas (HPA) localization data. **D** Fraction of genes in protein modules annotated as candidate, ciliary gene, or known ciliopathy gene. Only protein modules containing a fraction of candidate genes, ciliary genes, or ciliopathy genes above 0.6 are shown. Two sample t-tests were used for statistics. Prop., propagation; Expr., expression; PKA, protein kinase A

To test the model, we selected six ciliopathies not used for training, with at least ten known disease genes per ciliopathy. Combining the permuted network propagation scores with the mouse phenotype ranking significantly increased the precision-recall AUC (PR AUC) (Fig. 4a) and partial AUROC (0.95–1.0 specificity) (Fig. 4b), compared to the separate scores alone. The best model achieved a median partial AUROC of 0.94 across ciliopathies and all test rounds, with median values per round ranging from 0.91 to 0.96. We found limited differences for the AUROC, likely due to the small fraction of positive data points (Additional file 2: Fig. S8a). We observed performance differences between the different ciliopathies, but combining the two scores yielded the best result in most cases (Additional file 2: Fig. S8b). Interestingly, the gene expression score worsened the performance for most ciliopathies. This is likely due to the limited specificity of expression patterns across ciliopathy gene sets, while some, like CRD, showed distinct expression in photoreceptor cells, most other ciliopathies did not show such cell type or tissue specific signatures (Additional file 2: Fig. S7a). For predicting candidate genes for each ciliopathy individually, our gene expression model was not specific enough. An exception was CRD, where the addition of expression data improved prioritization (Additional file 2: Fig. S8b). This is likely due to the strong cell type-specific expression of retinal genes, suggesting that our gene expression model is biased toward retinal ciliary genes. Therefore, we proceeded with the model only combining the permuted network propagation and mouse phenotype ranking features (Additional file 1: Table S8). We also compared the performance of our final model to the scoring method from CilioGenics [48]. Our approach utilizes specified statistical scores to highlight phenotype-specific genes, whereas CilioGenics gives out scores for broader ciliary gene prediction. When applied to ciliopathy-specific gene prioritization, CilioGenics showed lower performance for most of the six tested ciliopathies (Additional file 2: Fig. S9), reflecting these methodological differences and the complementary focus of the two approaches.

To analyze which genes were highly ranked using our model, we performed gene set enrichment analyses (GSEA) after excluding the known disease genes from the ranking. We tested enrichment across several gene sets, including genes associated with ciliopathies in the databases but with low confidence scores (termed “weak ciliopathy genes”), known ciliary genes (from CiliaCarta [49] and SCGSv2 [50]), and protein localization data from HPA. High-ranking genes for all ciliopathies were enriched for “weak ciliopathy genes”, known ciliary genes, and proteins localized to microtubules and centrosomes, suggesting that our model effectively predicted cilia-related genes (Fig. 4c). However, not all ciliary genes were highly ranked, indicating that our model makes some separation between ciliary genes likely involved in ciliopathies and those that are not (Additional file 2: Fig. S10a), potentially reflecting essential genes or genes causing disorders not included in our analysis. In addition, the model preserved phenotype specificity, as gene rankings for related ciliopathies were correlated (Additional file 2: Fig. S10b).

We also investigated whether the protein modules obtained from clustering the PPI network were enriched for known ciliopathy genes, candidate genes (top 100 per ciliopathy), or ciliary genes (Fig. 4d). Most modules containing candidate genes had a high proportion of known ciliopathy genes. However, we also identified some modules containing candidate genes that had low fractions of known ciliopathy genes, such as those related to localization to telomeres, protein kinase activity, and Hedgehog signaling. Additionally, we identified modules with a high fraction of ciliary genes but a low fraction of known or candidate ciliopathy genes, such as those related to calcium entry and left/right asymmetry. The most promising candidate genes are likely within modules with a high fraction of known ciliopathy genes, as these modules are already linked to ciliopathies. However, genes in other modules could reveal more novel biology.

### Network propagation identifies *CEP43* as potential disease gene for individuals with ciliopathies

Using our model, we generated a list of 285 unique candidate genes highly ranked for various ciliopathies (Additional file 1: Table S9). We next sought to identify ciliopathy patients with variants in these genes among those with unsolved genetic diagnoses. First, we searched a cohort of 75 patients with BBS (38 patients), JBTS (13), or multi-systemic primary ciliopathies (24) from the Genomics England (GEL) 100,000 genomes database [51] for homozygous and compound heterozygous variants in the 285 candidate genes. We only considered variants with an allele frequency in GEL below 0.05% that were predicted to be deleterious based on variant effect predictors (VEPs), including CADD [52], ESM1b [53], AlphaMissense [54], and spliceAI [55].

**Fig. 5:**
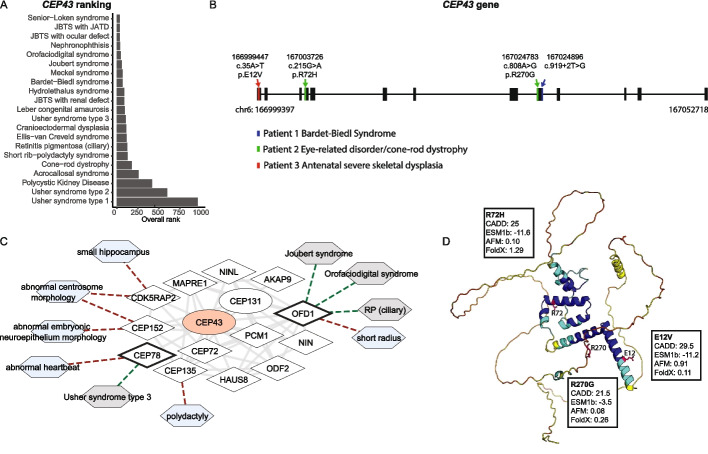
*CEP43* as novel ciliopathy disease gene. **A** Overall rank of *CEP43* for ciliopathies. **B** Schematic representation of the *CEP43* gene, highlighting variants identified in three patients, annotated with their clinical diagnoses. Variants are homozygous in patients 1 (blue) and 3 (red). **C** Protein module including CEP43, filtered to show proteins directly connected to CEP43. Genes in CiliaCarta and SCGSv2 are represented as diamonds, other genes as ellipses, and traits as hexagons. Known ciliopathy genes have a black border and CEP43 is visualized in red. Mouse phenotypes are colored blue and human ciliopathies grey. Solid edges represent protein interactions from Intact or STRING, with transparency indicating evidence scoring. Dotted edges denote gene-trait connections (red to mouse phenotypes and green to human ciliopathies). **D** Predicted protein structure from the AlphaFold Database [56] of CEP43 showing coding variants from (**B**), annotated with their CADD, ESM1b, AlphaMissense, and FoldX scores

Among these 75 individuals, we identified one patient with homozygous rare variants in *CEP43*, also known as *FGFR1OP* or *FOP*, which our method ranked in the top 100 highest ranked candidate genes for multiple ciliopathies (Fig. 5a). A follow-up phenotype-agnostic search in the entire GEL database for individuals with bi-allelic *CEP43* variants identified one more individual harboring likely pathogenic *CEP43* variants who also presented a clinical diagnosis related to primary ciliopathies. Finally, a third individual was identified in an independent cohort consisting of mixed diagnoses in consanguineous families (Fig. 5b, Additional file 1: Table S10). *CEP43* was highly ranked by our model based on its known interactions with proteins involved in human ciliopathies and mouse phenotypes closely related to ciliopathies (Fig. 5c).

The first individual identified among the 75 patients from GEL carried a diagnosis of BBS with postaxial polydactyly, mild progressive cone-rod dystrophy, congenital dysplasia of the hip, mild intellectual disability, and obesity (Fig. 5b, Additional file 1: Table S10). Diagnostic testing had been reported as being negative on the exit questionnaire by GEL. This individual carried a homozygous splice region variant c.919 + 2 (NC_000006.12:g.167024896 T > G) with a CADD score of 33 and a high predicted impact (spliceAI delta score = 0.99) (Fig. 5b). No other rare pathogenic variants were identified in known disease genes in this individual. The second individual from the larger GEL search had a diagnosis of retinal dystrophy of the CRD type with progressive visual loss and abnormalities of retinal pigmentation. This individual carried two heterozygous variants in *CEP43* (NC_000006.12:g.167003726G > A and NC_000006.12:g.167024783A > G) (Fig. 5b). Both variants have high predicted CADD scores and low minor allele frequencies (MAF) in gnomAD v4.1.0 [57] (0.0013% and 0.0082% respectively) but only p.R72H (NC_000006.12:g.167003726G > A) is predicted to be pathogenic by ESM1b and destabilizing by FoldX (Fig. 5d), while p.R270G (NC_000006.12:167024783A > G) is predicted to be a splice acceptor variant by spliceAI (delta score = 0.34). This individual also had a negative exit questionnaire by GEL after diagnostic testing but did harbor additional homozygous c.952G > A (p.Ala318Thr) variants in *CNGA3*. Lastly, we searched for *CEP43* variants in an independent cohort consisting of consanguineous families and identified a fetus with antenatal severe skeletal dysplasia (pregnancy terminated) with a homozygous p.E12V variant (NC_000006.12:g.166999447A > T, gnomAD MAF 0.000068%). This variant is predicted to be pathogenic by CADD, ESM1b, and AlphaMissense but not predicted to be destabilizing (Fig. 5d), suggesting another mechanism of pathogenicity. No other pathogenic variants in known disease genes were reported in this family following whole exome sequencing.

In previous literature, *CEP43* has already been functionally shown to be important for cilia assembly and disassembly, and a mutant mouse model displays skeletal phenotypes reminiscent of the fetus with skeletal dysplasia and *CEP43* variants identified in this work [58–62]. Based on these previous functional studies for CEP43 and on its interactions with known cilia-centrosome associated disease genes such as CDK5RAP2 or CEP135 (Fig. 5c), *CEP43* is a clear candidate gene for ciliopathies. Indeed, it had already been previously ranked at place 126 as a potential ciliary gene by CilioGenics [48] and is now also ranked among the top candidates for several ciliopathies by our model. We now identified three individuals with ciliopathy phenotypes (retinal, skeletal and neurological) harboring pathogenic variants in this gene. In addition to *CEP43*, a literature search revealed that three other high-ranking genes (*TTC26* [63], *SCLT1* [64] and *FUZ* [65]) (Additional file 1: Table S8) have already been associated with ciliopathies. Together, these observations support the utility of our model, combining network propagation scores with mouse phenotype data, in effectively prioritizing candidate disease genes for the discovery of likely genetic diagnoses, even in small cohorts of genetically unsolved ciliopathy patients.

## Discussion

Our study provides a framework for studying disease etiology and genotype-phenotype associations in rare diseases. As proof of principle, we show that network propagation scores contain phenotypic information for primary ciliopathies, as these scores improved retrieval of disease pairs with similar clinical phenotypes compared to the overlap in known disease genes. We also obtained insights into protein groups underlying these phenotypes upon clustering of the PPI network. Similarly, we show that the same approach can be used to identify mouse phenotypes related to rare diseases based on their genetics and that these similarities relate to clinical phenotypes. Furthermore, genetic knowledge of these mouse phenotypes improved prioritization of disease genes.

While ciliopathies are characterized by dysfunction of proteins linked to one organelle, there is a large variety in their clinical phenotypes. As ciliopathies are rare and genetically heterogeneous, it is challenging to assess how much phenotypic diversity can be explained purely by the disease-causing genes themselves. In our analysis, we have identified phenotype-specific protein modules which may facilitate the identification of ciliary pathomechanisms linked to specific clinical phenotypes. For primary ciliopathies, specificity was found in protein modules related to visual, hearing, renal, and skeletal phenotypes. These organ-specific protein modules also contained genes associated with multi-systemic ciliopathies. This could suggest that individuals with multi-systemic ciliopathies and mutations in genes within these modules are more likely to develop involvement of the corresponding organ. For instance, the module ‘cilium assembly – kidney development’ was enriched for ciliopathies with a renal phenotype, including NPHP, PKD, and SLS. Almost half of the mouse phenotypes enriched for this module were also related to the renal/urinary system. In addition, the skeletal ciliopathy SRP was associated with this module via Nek1. As a Nek1 mouse model has a renal phenotype [66] and some SRP subtypes also have renal involvement, this can suggest that patients with *NEK1*-associated SRP may be more likely to develop kidney disease than those with SRP caused by other genes. Such suggested associations must now be investigated in human ciliopathy cohorts, whereby the small number of affected patients may limit the analysis.

For genotype-phenotype associations, we were also particularly interested in JBTS, as multiple clinical subtypes of this disease have been described. When including the mouse phenotypes, JBTS with the skeletal phenotype JATD clustered together with the skeletal ciliopathies CED and SRP, indicating similar underlying mechanisms. One possibility to explore could be an abnormal centrosome morphology, as mice with this cellular phenotype cluster together with the skeletal human ciliopathies. The subtypes of JBTS with renal defect and JBTS with ocular defect did not specifically cluster with clinically similar human ciliopathies or renal/ocular mouse phenotypes, which could indicate that the current known subtype-associated genes are not specific enough to the clinical phenotype. Still, proteins associated with pure JBTS which clustered with proteins causing a specific subtype might be interesting targets for further studies into JBTS subtypes. For example, the protein module related to centriole replication contained proteins associated with pure JBTS and JBTS with JATD, suggesting that genes in this module, known to cause pure JBTS, such as *CEP120* and *KIAA0753*, may also cause JBTS with JATD. Since both genes have now also been involved in ciliopathies with skeletal phenotypes [34, 35], this supports our genotype-phenotype association.

In addition to organ-specific protein modules, we also identified protein modules associated with a large variety of human ciliopathies and mouse phenotypes. For instance, the module termed ‘BBSome, core’ was associated with various human ciliopathies, including BBS, and could not be linked to a specific clinical phenotype. However, these protein modules can still give more insights into molecular mechanisms. We identified a strong connection between proteins from the CPLANE complex, consisting of INTU, FUZ, WDPCP, and CPLANE2, and the BBSome core proteins. *WDPCP* has already been identified as a BBS gene [67], but the other proteins have not been clearly linked to the BBSome. A recent study showed that FUZ might link GPR161 to the BBSome for removal from cilia [68], also suggesting a link between FUZ and the BBSome. In further studies, the links between the CPLANE complex, other proteins identified here in the BBSome core protein module, such as RFX1, TBC1D32, and ARL13A, and the BBSome should be investigated. Another module associated with BBS showed its connection to protein polyubiquitination which aligns with previous papers linking ubiquitination and the BBSome [41–43]. However, the strength of the association between the BBS gene *TRIM32,* which is part of this module, and BBS requires further evaluation [39].

Furthermore, we showed that genetic knowledge can be transferred from other groups of traits to rare diseases. It has already been shown that GWAS traits share genes with ciliopathies [42] and that mouse models can help ciliopathy gene prediction [23]. Here, we demonstrate that network propagation scores can systematically rank mouse phenotypes based on their relevance to the corresponding rare disorder. Disease gene recovery based on mouse phenotypes was not equally accurate for all human ciliopathies, suggesting that some high-ranked mouse phenotypes are related to the corresponding human ciliopathy primarily due to shared known genes, while other genes associated with the mouse phenotypes do not provide additional insights into human ciliopathies. Additionally, this variability can be explained by differences in the resolution and representation of relevant phenotypes among the approximately 3,500 available mouse phenotypes. Interestingly, the retrieval of human ciliopathy genes using randomly selected mouse phenotypes still outperformed random gene selection, suggesting a potential bias in the available mouse models or the protein interaction network. This bias may arise because human disease genes and genes used in mouse models tend to be more interconnected within protein interaction networks.

Combining information from mouse phenotypes with network propagation scores enabled prioritization of ciliopathy-specific disease genes with greater accuracy than general ciliary gene predictions, such as those from CilioGenics [48]. This highlights the strength of our framework for disease-specific gene prioritization, while other approaches provide a broad resource for general ciliary gene discovery and can be used in a complementary manner. Our framework led to the identification of likely pathogenic variants in *CEP43*, also known as *FGFR1OP* or *FOP,* in three individuals with primary ciliopathy syndromes, including a BBS-like phenotype, a severe skeletal dysplasia, and a retinal phenotype. Further supporting the utility of our approach for ranking and identifying candidate ciliopathy genes, several genes that were not part of our seed genes (based on the databases used) and that our method listed as highly ranked candidate ciliopathy genes have been found to be associated with ciliopathies in the meantime, including *TTC26* [63], *SCLT1* [64] and *FUZ* [65].

Even though our study is helpful in prioritizing ciliopathy candidate genes, the identification of novel ciliary genes in general might be limited. As our analysis is based on known protein interactions, the bias of current research will be reflected in our results. Furthermore, the efficiency of the method strongly relies on the reliability of the databases used. As mentioned above, several disease genes were not listed in the databases that we used to determine the seed genes for the network propagation, as databases are not always up-to-date. Furthermore, the quality of the data available in the primary literature, with respect to gene-phenotype correlations, is very variable and clinical descriptions are often incomplete in publications. Lastly, our analysis is limited by the generalization of the interactome. Given that the ciliome is dynamic across developmental stages and tissues [69–71], incorporating tissue-, development-, or cilium-specific interaction networks could enhance our analysis and reduce false positive interactors in certain contexts. Future studies should further refine emerging methods for assessing tissue-specific gene effects to better capture the heterogeneity of the ciliome and its dynamic interactome [72–74]. In our study, the limited tissue-specificity of ciliopathy gene expression constrained the model’s predictive power, except for retinal genes where distinct patterns enhanced prioritization, highlighting the need for more refined, context-aware data integration.

## Conclusions

We performed a systematic analysis of rare disease etiology and phenotype-specific protein modules in primary ciliopathies. We identified mouse phenotypes that give a good representation of ciliopathies, which can be used for further studies of disease mechanisms. We also employed these models to prioritize ciliopathy-specific candidate genes and identified novel likely pathogenic variants in three ciliopathy patients. In this study, we focused on ciliopathies, but this approach can be used to study etiology and genotype-phenotype associations in a large set of rare disorders.

## Methods

### Network propagation for ciliopathies and mouse phenotypes

For the PPI network, we used the Open Targets Interactome Network (release 22–07–2022). This network integrates direct (i.e., physical) and indirect interactions from IntAct, SIGNOR, Reactome, and STRING (scores > = 0.4). Duplicated edges and self-loops were removed, resulting in a network containing 19,306 nodes and 1,215,355 edges.

Seed genes for ciliopathies were obtained from the Open Targets genetics portal, selecting those with gene-trait evidence scores > 0.5 for Genomics England PanelApp, Orphanet, Gene2Phenotype, UniProt literature, UniProt variants, ClinGen, and Gene Burden, and > 0.8 for ClinVar. Genes were curated to avoid overlap between subtypes of a disorder and to include recently identified genes. This resulted in the selection of 21 primary ciliopathies with at least two seed genes each. For mouse phenotypes, seed genes were also obtained from the Open Targets genetics portal as human orthologs, requiring a minimum of 10 seed genes per phenotype to exclude mouse models specific to only one monogenic disease. In total, 3,524 mouse phenotypes were included in the analysis.

We performed network propagation for ciliopathies and mouse phenotypes using the Personalized PageRank algorithm from the R package igraph v1.3.2, utilizing the same PPI network based on the human interactions. Seed genes were assigned a weight of 1.

### Comparison of ciliopathies

We calculated the distance between ciliopathies through two methods. As a first method, the seed genes were used as binary input for computing the Jaccard distance between diseases using the R package stats v4.2.1. As a second method, z-scored network propagation scores for all 19,306 proteins were used as input for computing Euclidean distances between diseases. Using these distances, we hierarchically clustered the ciliopathies with Ward D2 linkage and compared the distances between ciliopathies with shared phenotypes by calculating the ROC curves and the AUROC. Ciliopathies were manually annotated with the following phenotypes: retinal defects, hearing loss, CNS defects, polydactyly, renal defects, and skeletal defects. We calculated the ROC curves and AUCs by considering ciliopathy pairs with at least one shared clinical phenotype as positives, as well as by taking each clinical phenotype separately. 95% confidence intervals were calculated using the ci.auc function from the R package pROC v1.18.5 which uses 2000 stratified bootstrap replicates.

### PPI network clustering

We clustered the PPI network using the Walktrap clustering algorithm from the R package igraph v1.3.2. Given that ciliary complexes are expected to be small, we performed re-clustering until every protein module consisted of < = 20 proteins or until 5 rounds of re-clustering were completed. We observed that protein modules with more than 20 proteins after 5 re-clustering rounds did not continue to split with further clustering attempts. To associate protein modules with ciliopathies and mouse phenotypes, we evaluated whether network propagation scores were significantly higher in the respective protein module compared to the full network using Wilcoxon Rank Sum tests. After *p*-value adjustment across all protein modules for each ciliopathy, we selected those with a *p*-value < 0.05 and at least one trait-related seed gene as associated with the trait. For naming of the protein modules, we conducted GO enrichment analysis combined with manual annotation. For visualization, we used Cystoscape [75] and annotated nodes with genes from SYSCILIA Gold Standard v2 (SCGSv2) [50] or CiliaCarta [49] as ciliary and color-coded genes based on their presence in ciliary protein complexes identified by Boldt et al. [36].

### Mouse phenotype clustering with ciliopathies

To compare mouse phenotypes with human ciliopathies, we calculated the Euclidean distance between the ciliopathies and all mouse phenotypes using scaled and centered network propagation scores. For each human ciliopathy, the 20 mouse phenotypes with the shortest distance were selected. We compared the selection of mouse phenotypes using network propagation scores to selection using the Jaccard index of the overlap in seed genes. In addition, we assessed the robustness of both approaches by randomly removing a increasing fraction of human ciliopathy seed genes before calculating the distances between network propagation scores and the seed gene overlap with the mouse phenotypes. Subsequently, we performed hierarchical clustering of the human ciliopathies with these selected mouse phenotypes using Euclidean distance and Ward D2 linkage. The number of clusters was determined to be 11 based on the optimal separation of human ciliopathies within the clusters. Mouse phenotypes were then annotated with their ancestor term and associated with protein modules. For visualization, the ancestor terms and protein modules obtained from the network clustering with the highest fraction within each cluster were selected.

### Benchmarking ciliopathy gene prediction with mouse phenotype ranking

To test whether mouse phenotypes can contribute to candidate gene prediction for ciliopathies, we selected varying numbers of mouse phenotypes with the shortest Euclidean distances to each ciliopathy. Subsequently, we removed the known disease genes of the respective ciliopathies from the seed genes of the selected mouse phenotypes and reran network propagation. Gene ranks from the selected mouse phenotypes were then combined using the geometric mean of their network propagation scores, and ROC curves and AUROCs were calculated with known disease genes as positives. We tested this for different numbers of mouse phenotypes between 10 and 100 and also for Euclidean distance thresholds between 160 and 190, and found that 10 mouse phenotypes were optimal for recovering ciliopathy genes (Additional file 2: Fig. S4). For comparison, we applied the same pipeline with random mouse phenotypes and evaluated AUROC differences using a t-test. In addition, we assessed the recovery of known disease- causing genes by selecting mouse phenotypes using seed gene overlap instead of the similarity in network propagation scores. After selection of mouse phenotypes, known human ciliopathy genes were again removed from their seed genes and network propagation scores were calculated to generate gene rankings. Finally, to further evaluate the contribution of network propagation to both mouse phenotype selection and gene ranking, we performed an additional test on ciliopathies with at least five seed genes. In each case, we removed 60% of the genes for testing with 10 random test/train splits. Mouse phenotype gene sets were kept intact and not split. In each round, the top 10 mouse phenotypes were selected either by network propagation scores calculated using the training genes or the Jaccard index overlap in seed genes. Gene ranking was then carried out either by aggregating propagation scores across the selected phenotypes using the geometric mean or by counting the frequency of seed gene occurrences. AUROCs were calculated for each approach to compare their performance.

### Gene expression of ciliopathy genes

To analyze the gene expression of ciliopathy genes, we obtained single-cell RNA sequencing data from HPA and centered and scaled the data across cell types. The data was split into a test and training set, with the test set containing 10% of known ciliopathy genes and 10% of all other genes, and the training set containing the remaining 90% of all genes. First, we performed an ANOVA test with the training data to identify cell types with significantly different expression levels of known ciliopathy genes compared to the other genes. Cell types with a *p*-value < 0.01 were selected as features for a logistic regression model. The model’s weights were determined using the training data, with known ciliopathy genes as positives and the other genes as negatives. Finally, we evaluated the model by calculating the ROC curve and AUROC using the test data, treating the known ciliopathy genes as positives.

### Training and testing of model for candidate gene prioritization

To develop a model for prioritizing genes associated with different ciliopathies, we trained a logistic regression model incorporating three features: mouse phenotype ranking, network propagation *p*-values, and gene expression data. We selected four ciliopathies, each with at least 20 seed genes, to obtain model weights. For each ciliopathy, we performed multiple rounds of calculating separate feature scores based on five training genes, while the remaining disease genes were reserved as positives for the overall model. The number of rounds was determined by the total number of seed genes, ensuring that each gene was used only once in the training rounds. As negative controls, 150 genes not associated with the respective ciliopathy were randomly selected per round.

Network propagation scores were computed using the Personalized PageRank algorithm with the training genes as seed nodes. To prevent the high ranking of well-studied hub genes, we ran 1,000 permutations with random seed genes to compute *p*-values while maintaining the same PPI network. For ranking of genes by mouse phenotypes, we calculated the Euclidean distances between scaled propagation scores derived from the five training genes and the scaled mouse phenotype propagation scores. The 10 mouse phenotypes with the shortest distance to the ciliopathy were selected, and ranks for every gene were combined using the geometric mean. For the gene expression scores, a logistic regression model, as described above, was trained every round, excluding the test genes. The expression scores for each gene were then calculated using the trained model. All feature scores were min–max scaled to ensure they were in the same range.

After calculating the separate feature scores for each round and ciliopathy, the scores were aggregated to train one logistic regression model for all ciliopathies. This model used the test genes as positives and the randomly selected control genes as negatives, resulting in 727 positive genes and 3,643 negative genes. Separate models were trained with different combinations of the three features.

To evaluate our models for other ciliopathies, we selected six different ciliopathies with at least ten seed genes each. As with the training of the model, we used five seed genes as training genes and the remaining genes as test genes. In addition, 1,000 genes were randomly sampled as controls. Separate scores were again calculated using the training genes and combined using the trained models. For each ciliopathy, ten rounds of testing were run with different train/test splits for the genes. The overall score was then used to calculate the PR AUC, partial AUROC with specificity between 0.95 and 1.0, and the AUROC. These metrics were compared between the different models to determine the optimal combination of features. This model was then used to calculate the final gene ranking for each ciliopathy, using all seed genes to calculate the separate feature scores.

### Assessment of gene prioritization

To gain insight into our gene prioritization, we conducted GSEA using various gene sets on the gene ranking excluding the known disease genes. Genes with gene-trait evidence scores below the thresholds defined for the seed genes were categorized into the ‘weak evidence’ gene set. In addition, genes from SCGSv2 and CiliaCarta were defined as ciliary genes. We also used localization data from HPA as gene sets. We performed GSEA for each ciliopathy separately and then counted the number of ciliopathies for which the gene sets (max size = 2000) were significantly (Benjamini–Hochberg adjusted *p*-value < 0.05) enriched among their high-ranking genes.

Furthermore, we examined the presence of high-ranking genes within protein modules. For each ciliopathy, we selected the top 100 ranked genes, excluding all seed genes. We calculated the fraction of these candidate genes in each protein module. As a comparison, we also calculated the fraction of ciliary and known disease genes in each module, aggregated across all ciliopathies.

Lastly, we compared our gene rankings with the ranking of genes based on the CilioGenics score. The score was downloaded and the AUROCs were calculated for the six ciliopathies used to test our approach. As the CilioGenics score reflects general ciliary gene prediction rather than being specific to individual ciliopathies, the same gene ranking was used for all six disorders.

### Variant identification in patient cohort

A cohort of 75 patients with BBS (38 patients), JBTS (13), and multi-systemic ciliopathies (24) was collected from the rare disease cohort in the Genomics England database. We scanned this cohort for homozygous and compound heterozygous variants in top candidate genes obtained from the gene prioritization and scanned these for their allele frequency and CADD scores. Among the findings, compound heterozygous variants in *CEP43* identified in an individual with a diagnosis of BBS, were found to be of particular interest. Following this discovery, we searched the broader Genomics England Database for variants in *CEP43*, leading to the identification of one more individual with an eye-related disorder carrying two mutations in *CEP43*. Lastly, a third individual was identified in a Saudi Arabian cohort through targeted reanalysis of existing sequencing data. For all coding variants, CADD, ESM1b, AlphaMissense, FoldX, and spliceAI scores were obtained to assess their potential impact. In addition, the AlphaFold prediction of CEP43 was retrieved from the database. Direct interactors of CEP43 and their association with human disorders and mouse phenotypes were retrieved and visualized to provide further context to the identification of *CEP43* as candidate gene.

## Supplementary Information

Additional file 1: Tables S1-S10.

Additional file 2: Fig. S1-S10.

## Data Availability

Code is freely available on [https://www.github.com/eaarts/networkPropagation] and on Zenodo with DOI: [10.5281/zenodo.17633403] under the MIT license [76]. The network propagation and all subsequent analysis were performed using R software (v.4.2.1) as described in the methods. The following packages were used: igraph (v.1.3.2, for Personalized PageRank and walktrap clustering), pROC (v.1.18.0, for ROC curves and AUCs calculations when applicable), clusterprofiler (v.4.4.4, for GOBP enrichment analysis in the description of the modules as well as GSEA tests), ComplexHeatmap (v.2.12.1, for heatmap visualization), ggplot2 (v.3.4.0 for plotting), circlize (v.0.4.15) and RColorBrewer (v.1.1.3) both for color palette generation. sparklyr (v.1.7.8) and sparklyr.nested (v.0.0.3) both to obtain datasets, dplyr (v.1.1.4) and tidyverse (v.1.3.2) both to organize code, doParallel (v.1.0.17) and foreach (v.1.5.2) both for parallelization, dendextend (v.1.15.2 for dendogram plotting), ggpubr (v.0.5.0 for calculating statistics in plots), and org.Hs.eg.db (v.3.15.0 for gene ID mapping). All data generated or analyzed during this study are included in this published article (and its Supplementary files). Publicly available repositories can be accessed as follows: OTAR interactome ([https://ftp.ebi.ac.uk/pub/databases/opentargets/platform/22.09/output/etl/parquet/interaction/]) [28, 77], Open Targets Genetics portal ([https://genetics.opentargets.org/]) [77, 78], Experimental Factor Ontology v.3.60.0 ([https://www.ebi.ac.uk/efo/]) [79, 80], CiliaCarta ([https://tbb.bio.uu.nl/john/syscilia/ciliacarta/]) [49, 81], SCGSv2 ([http://syscilia.org/]) [50], and HPA ([https://www.proteinatlas.org/]) [82, 83]. Research on the de-identified patient data used in this publication can be carried out in the Genomics England Research Environment subject to a collaborative agreement that adheres to patient led governance. All interested readers will be able to access the data in the same manner that the authors accessed the data. For more information about accessing the data, interested readers may contact research-network@genomicsengland.co.uk or access the relevant information on the Genomics England website: [https://www.genomicsengland.co.uk/research]. All data generated or analyzed during this study are included in this published article (and its Supplementary files). Publicly available repositories can be accessed as follows: OTAR interactome ([https://ftp.ebi.ac.uk/pub/databases/opentargets/platform/22.09/output/etl/parquet/interaction/]), Open Targets Genetics portal ([https://genetics.opentargets.org/]), Experimental Factor Ontology v.3.60.0 ([https://www.ebi.ac.uk/efo/]), CiliaCarta ([https://tbb.bio.uu.nl/john/syscilia/ciliacarta/]), SCGSv2 ([http://syscilia.org/]), and HPA ([https://www.proteinatlas.org/]). Research on the de-identified patient data used in this publication can be carried out in the Genomics England Research Environment subject to a collaborative agreement that adheres to patient led governance. All interested readers will be able to access the data in the same manner that the authors accessed the data. For more information about accessing the data, interested readers may contact research-network@genomicsengland.co.uk or access the relevant information on the Genomics England website: [https://www.genomicsengland.co.uk/research].
