# Supplementary material for: Network-based framework for studying etiology and phenotypic diversity in primary ciliopathies
Source: Genome Biol. 2026 Feb 19;27:105. doi: 10.1186/s13059-025-03899-7 (PMC13020049; doi:10.1186/s13059-025-03899-7)
Supplement: Supplementary file 2 — Additional file 2: Fig. S1-S10. [file 13059_2025_3899_MOESM2_ESM.pdf]

Supplementary information

Network-based framework for studying etiology and phenotypic diversity in primary ciliopathies

Ellen M. Aarts[1], Diederik S. Laman Trip[1], Ruxandra Neatu[2], Charlotte G. Martin[2], Beth Riley[2], Alison Kraus[3], Abigail Green[3], Mohamed H. Al-Hamed[4], Rachel E. Armstrong[2], John A. Sayer[2,5,6], Ruxandra Bachmann-Gagescu[7,8,*], Pedro Beltrao[1,*].

[1] Institute for Molecular Systems Biology, ETH Zurich, Zurich, Switzerland
[2] Bioscience Institute, Newcastle University, Newcastle Upon Tyne, UK
[3] Yorkshire Regional Genetics Service, Chapel Allerton Hospital, Leeds, UK
[4] Department of Clinical Genomics, Center for Genomic Medicine, King Faisal Specialist Hospital and Research Center, Riyadh 11211, Saudi Arabia
[5] Renal Services, The Newcastle upon Tyne Hospitals NHS Foundation Trust, Newcastle, UK
[6] NIHR Newcastle Biomedical Research Centre, Newcastle Upon Tyne, UK
[7] Institute of Medical Genetics, University of Zurich, Schlieren, Switzerland
[8] Department of Molecular Life Sciences, University of Zurich, Zurich Switzerland

* Shared last authorship. Correspondence to ruxandra.bachmann@mls.uzh.ch and pbeltrao@ethz.ch.

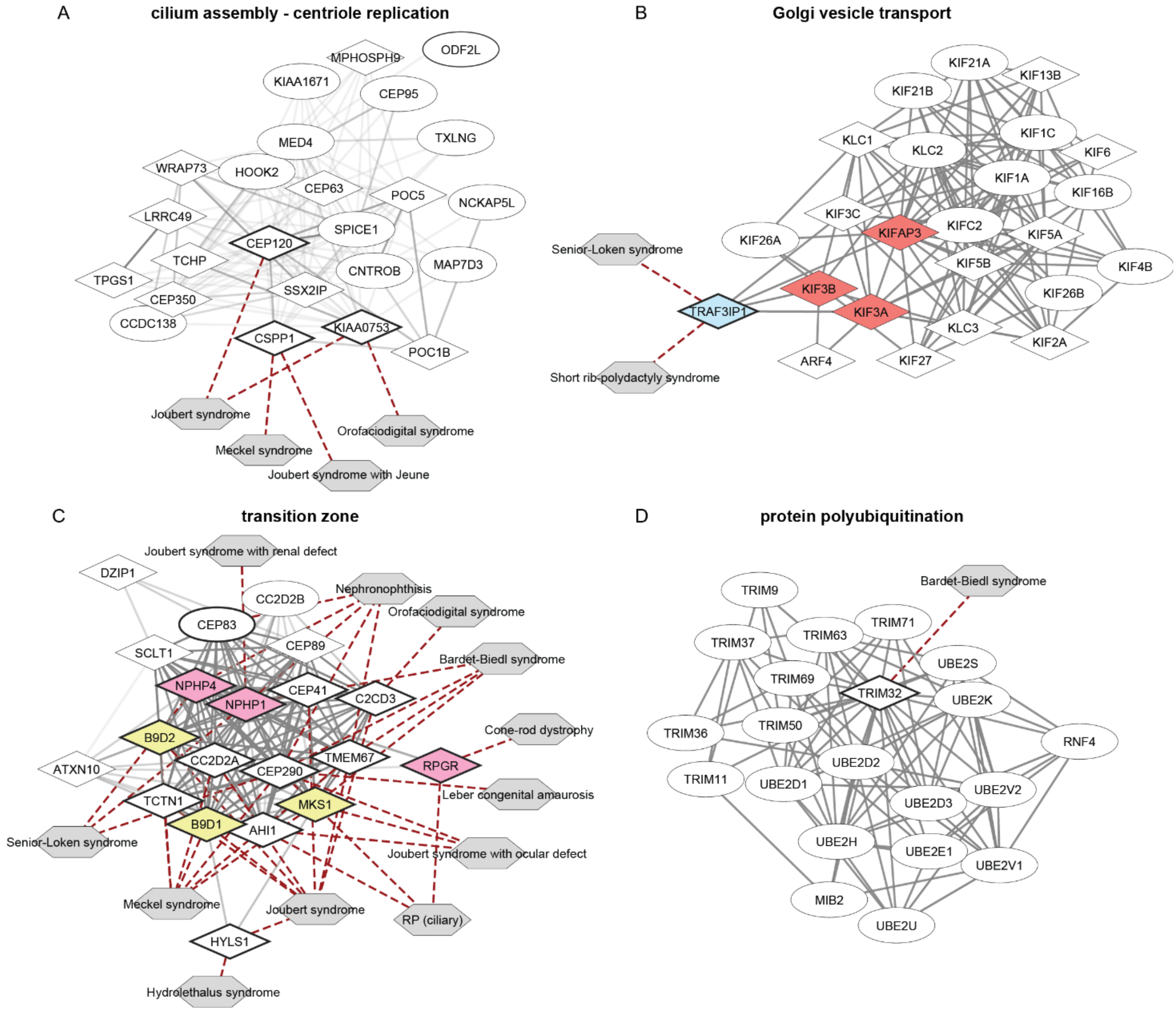

Fig. S1: Examples of protein modules associated with ciliopathies. *(A) Detailed view of the module "cilium assembly – centriole replication". (B) Detailed view of the module "Golgi vesicle transport". Only first-degree neighbors of TRAF3IP1 connected by edges with a score ≥0.92 were kept for visualization. Node colors by Boldt et al. (36): IFT-B1 (light blue) and KIF3 (red). (C) Detailed view of the module "transition zone". Node colors by Boldt et al.: RPGR (pink) and MKS1 (yellow). (D) Detailed view of the module "protein polyubiquitination". First- and second-degree neighbors of TRIM32 connected by edges with a score ≥ 0.92 were kept for visualization. Genes in CiliaCarta (44) and* SYSCILIA Gold Standard v2 (SCGSv2) *(45) are represented by diamonds, other genes by ellipses, and ciliopathies by grey hexagons. Known ciliopathy genes are outlined in black (as based on initial determination of ciliopathy seed genes). Solid edges represent interactions from Intact or STRING, with transparency indicating the evidence score. Dotted edges indicate gene-disease associations.*

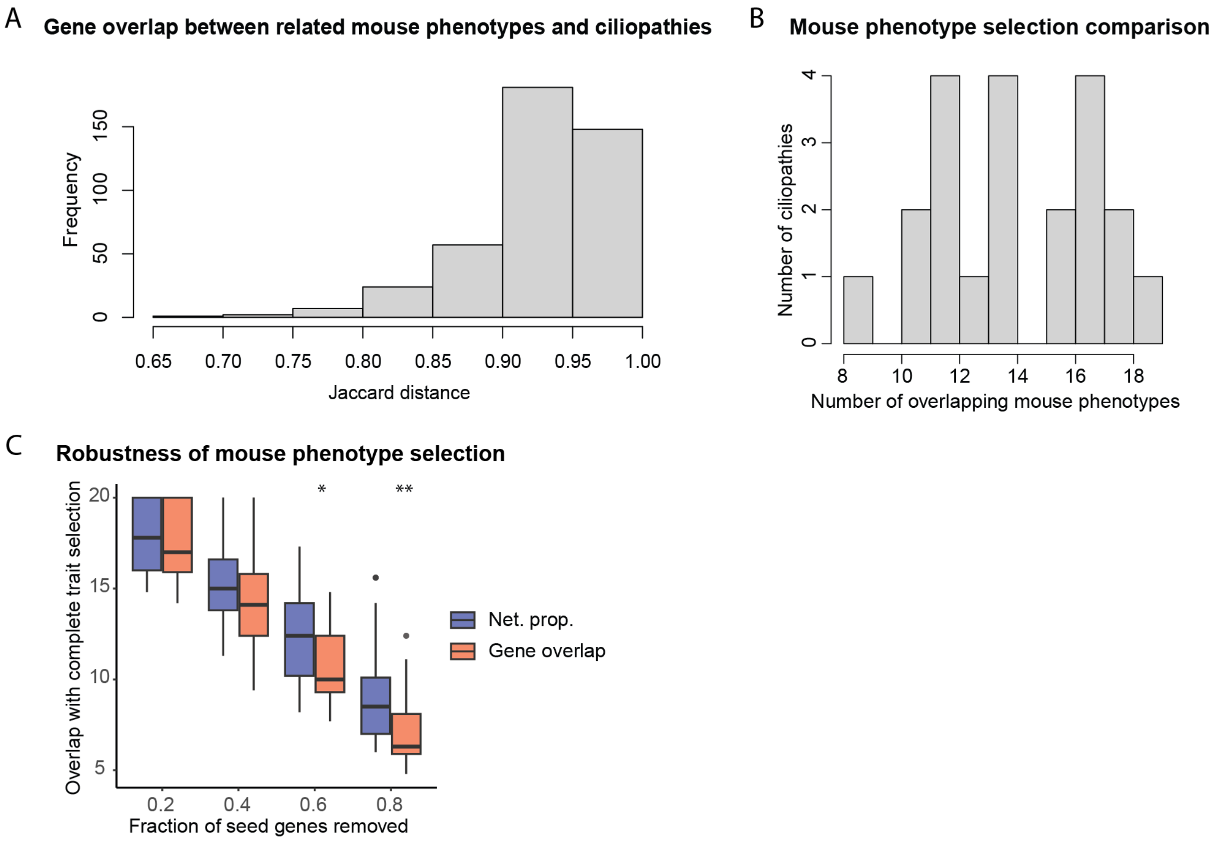

Fig. S2: Mouse phenotype selection is more robust using network propagation scores than seed gene overlap. *(A) Jaccard distances (1 – Jaccard similarity) between the top 20 mouse phenotypes selected using network propagation scores and their corresponding ciliopathy (420 comparisons: 20 phenotypes × 21 ciliopathies). (B) Number of overlapping mouse phenotypes (out of 20) between selections based on network propagation scores and those based on seed gene overlap. (C) Number of overlapping mouse phenotypes (out of 20) after progressively reducing the number of seed genes, comparing selections based on network propagation scores or seed gene overlap to those obtained using the full seed gene set. Two sample t-tests were used for statistics: * < 0.05 and ** < 0.01.*

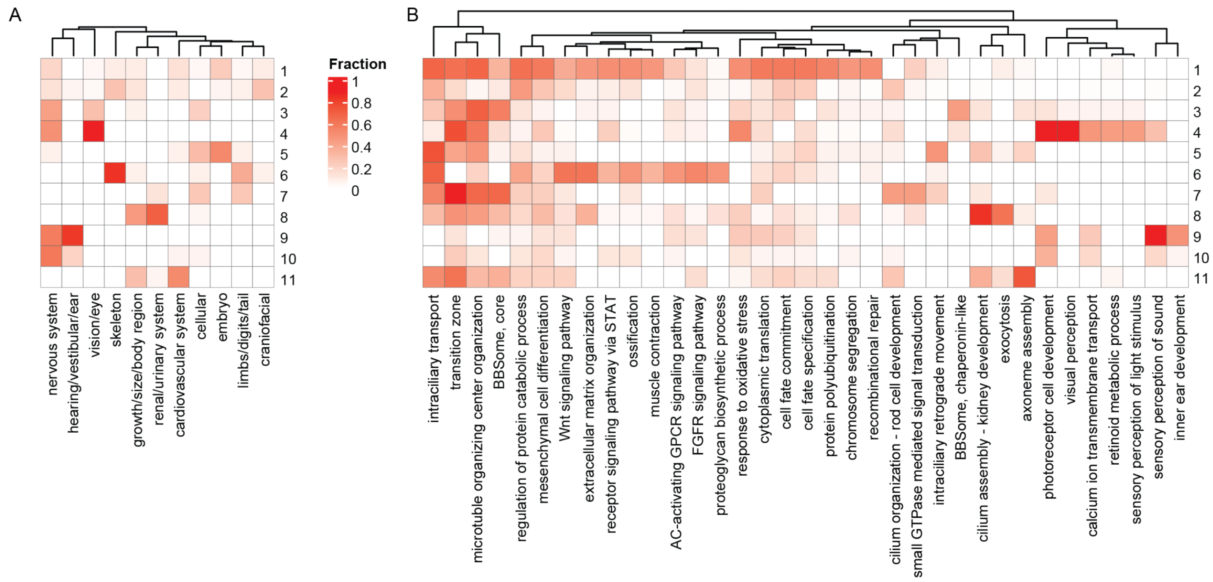

Fig. S3: Mouse phenotype and human ciliopathy clusters associate to specific global phenotypes and protein modules. *(A) Fraction of mouse phenotypes within each cluster from fig. 3a belonging to respective global phenotype, i.e., ancestor. (B) Fraction of mouse phenotypes within each cluster from fig. 3a associated with the respective protein module.*

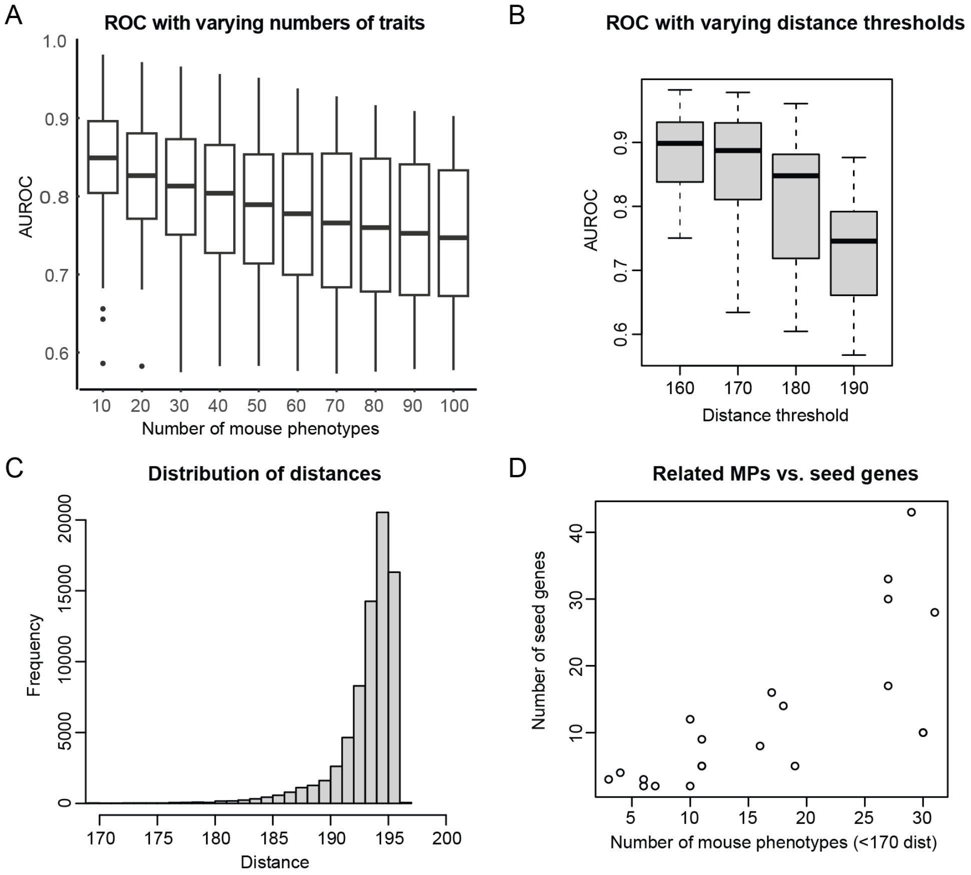

Fig. S4: Selection of mouse phenotypes based on distance thresholds. *(A) AUROC scores (median ± IQR) for prediction of human ciliopathy genes using varying numbers of top-ranked mouse phenotypes with the most similar network propagation scores to each human ciliopathy. Ten mouse phenotypes give the highest AUROC scores (median AUROC = 0.849) for retrieving known human ciliopathy genes. (B) Same as (A), but selection was based on Euclidean distance thresholds between network propagation scores instead of a fixed number of phenotypes. A threshold of ≥180 was required to obtain mouse phenotypes for all ciliopathies. This results in similar AUROC scores as with top 10 phenotypes (median AUROC = 0.848). (C) Distribution of Euclidean distances between human ciliopathies and all 3,524 mouse phenotypes in the Mouse Genome Database. Only 0.82% of distances are below 180. (D) Correlation between the number of seed genes for a ciliopathy and the number of mouse phenotypes within a distance cutoff of 170 (Pearson r = 0.74).*

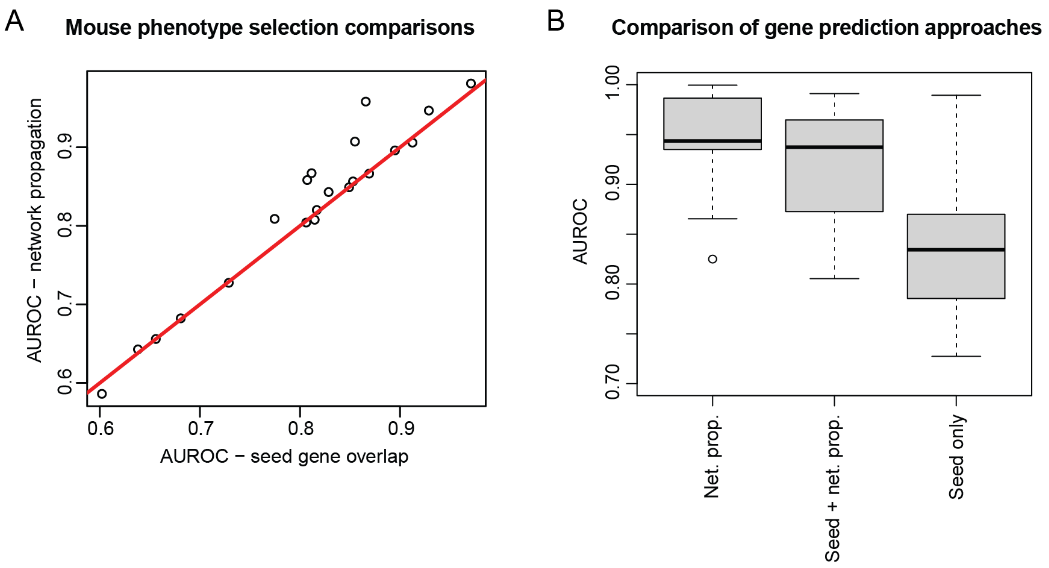

Fig. S5: Network propagation improves gene prediction from mouse phenotypes compared to gene overlap. *(A) Prediction of human ciliopathy genes based on top 10 mouse phenotypes selected either by network propagation scores or by seed gene overlap. Mouse phenotypes were first selected using the full seed gene sets, then known human ciliopathy genes were removed from the seed gene sets of the selected mouse phenotypes to rerun network propagation with the remaining genes, and the resulting scores were combined to rank candidate genes. Four ciliopathies show an increase in AUROC of at least 0.05. (B) Prediction of human ciliopathy genes using three approaches: 1) network propagation for both mouse phenotype selection and gene ranking (Net. prop.), 2) phenotype selection based on seed gene overlap followed by network propagation for ranking (Seed + net. prop., and 3) phenotype selection and ranking based only on seed gene overlap and seed gene counts (Seed only). AUROC scores were calculated for ciliopathies with at least five seed genes, using 40% of genes for training across 10 random train/test splits.*

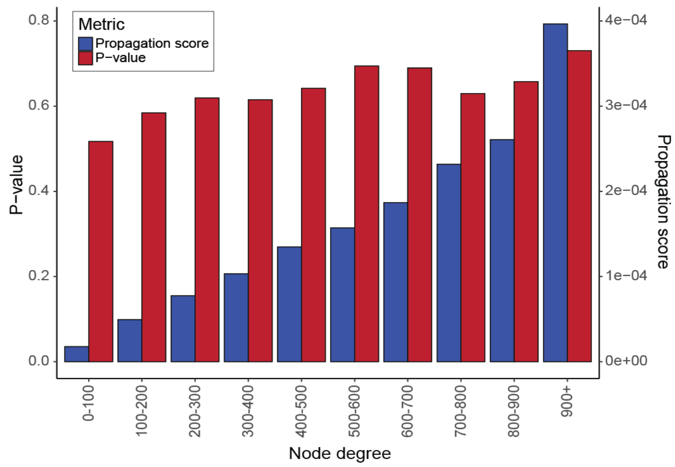

Fig. S6: Network propagation scores depend on node degree. *Per category of node degrees, the mean propagation scores (blue) and -log10 p-values (red) are visualized. P-values are obtained from permutation tests with randomly selected seed genes. Permutation removes correlation between node degree and network propagation scores.*

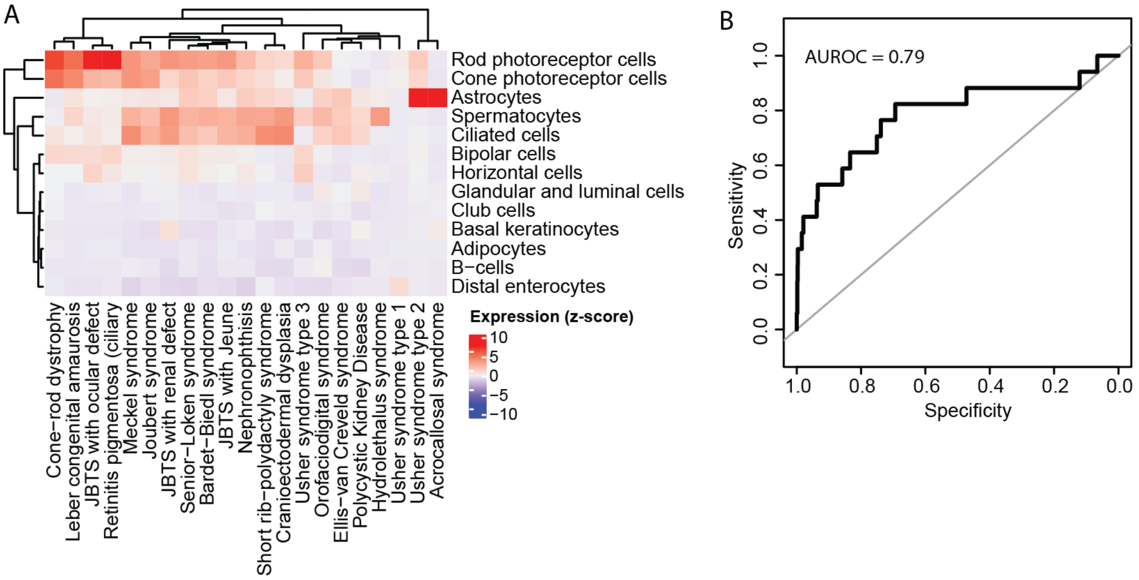

Fig. S7: Gene expression to predict human ciliopathy genes. *(A) Mean expression of ciliopathy-associated genes in selected cell types (z-scored over all cell types) obtained from HPA. Cell types were selected based on significant differential expression between known ciliopathy genes and other genes. (B) ROC curve for retrieval of human ciliopathy genes using a logistic regression model with gene expression in cell types from (A) as features.*

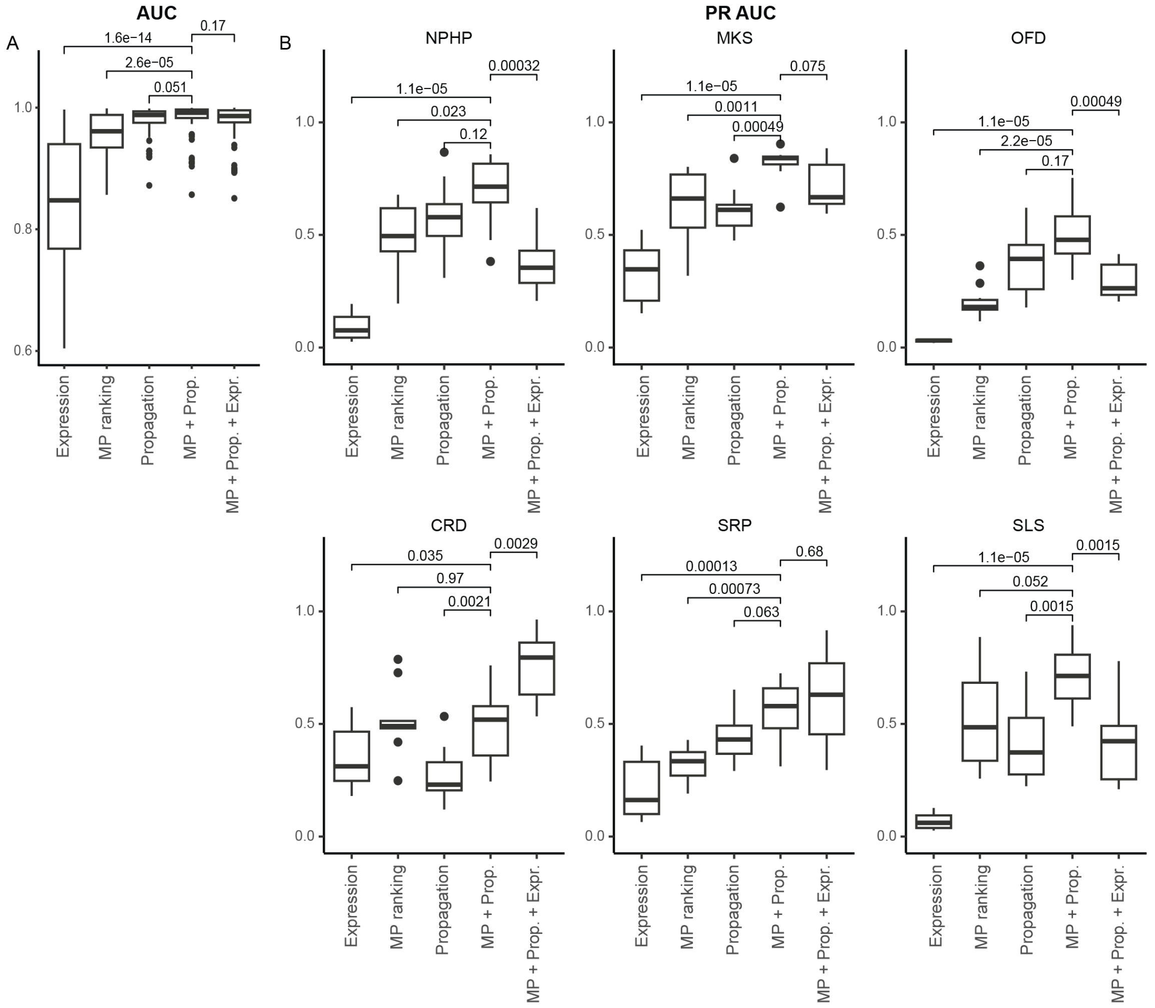

Fig. S8: Accuracies of ciliopathy candidate gene prediction models. *(A) AUC of models combining mouse phenotype ranking (MP), permuted network propagation scores (Propagation), and gene expression scores (Expression) in various combinations. (B) PR AUC of models in (A), split by ciliopathies used for testing the models. Barplots represent medium +/- IQR and t-tests for statistics.*

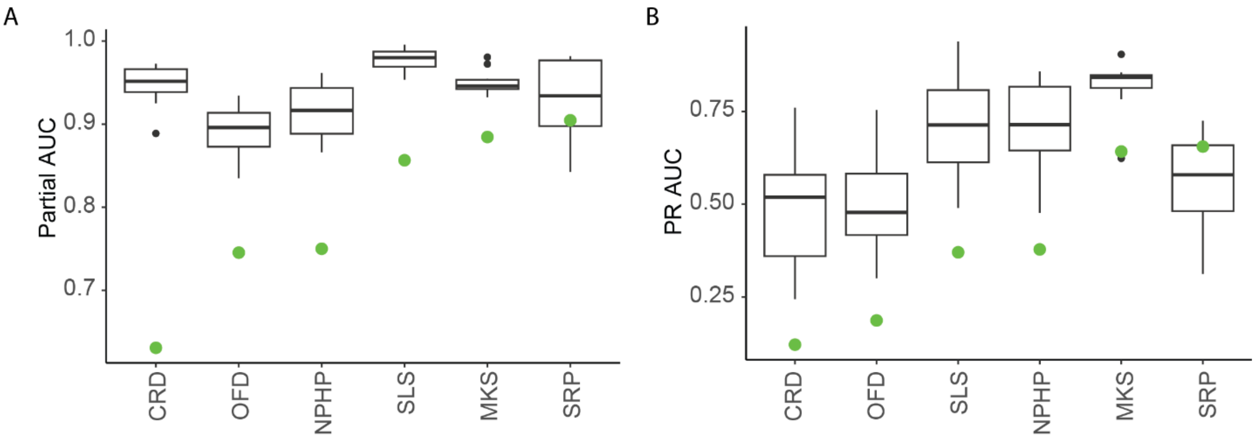

Fig. S9: Comparison of our model to CilioGenics for ranking ciliopathy disease genes. *(A) Partial AUC (specificity 0.95-1.00) and (B) PR AUC scores for retrieval of disease genes for six ciliopathies with at least ten seed genes. Green dots represent the partial AUCs and PR AUCs for the respective ciliopathies using CilioGenics scores for disease gene predictions. Barplots represent medium +/- IQR and t-tests for statistics.*

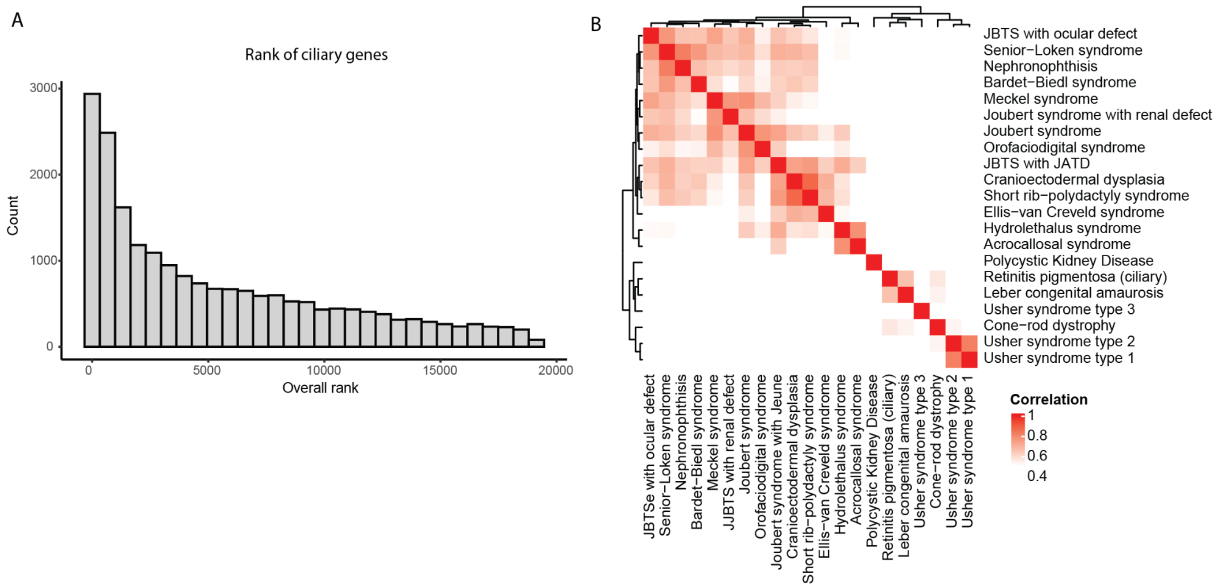

Fig. S10: Scoring of genes per ciliopathy. *(A) Overall rank from candidate gene prediction model of genes in CiliaCarta and/or* SCGSv2 (ciliary genes) *for 21 ciliopathies. Ciliary genes are more often ranked high but many are also ranked low. (B) Correlations of prediction scores between the 21 ciliopathies. The color scale starts at 0.5 to enhance visualization of correlation scores.*
